# Paradigm shifts in mental health legislation: a comparative analysis of WHO guidelines (2006 & 2023) and implications for future policies

**DOI:** 10.3389/fpsyt.2026.1804522

**Published:** 2026-03-10

**Authors:** Qian Xue, Bo Chen

**Affiliations:** 1 KoGuan School of Law, Shanghai Jiao Tong University, Shanghai, China; 2 Faculty of Law & Justice, The University of New South Wales, Sydney, NSW, Australia; 3 School of Law, Minjiang University, Fuzhou, China

**Keywords:** CRPD, mental health law and policy, mental wellbeing, paradigm shift, World Health Organization

## Abstract

This study presents a comparative analysis of the World Health Organization’s mental health legislative guidance spanning nearly two decades, documenting a fundamental paradigm shift that reflects broader transformations in psychiatric research and practice. By systematically examining the WHO’s Resource Book on Mental Health, Human Rights and Legislation (2006) and the Mental health, human rights and legislation: guidance and practice: Guidance and Practice (2023), we illuminate how the UN CRPD has reconceptualized mental health law. Our analysis reveals a decisive transition from biomedical to human rights-based approaches. The 2006 guidelines, while progressive for their time, accepted involuntary hospitalization under procedural safeguards and endorsed substitute decision-making mechanisms. In contrast, the 2023 framework fundamentally rejects coercion, promotes deinstitutionalization, universal legal capacity, and supported decision-making. This transformation directly addresses multiple themes central to contemporary psychiatric research: promoting mental wellbeing through supportive rather than coercive interventions; reimagining crisis response through peer support rather than restraint; and ensuring participation of persons with lived experience in policy making. Despite its ambition, the implementation of the 2023 Guidance faces some challenges. First, its commitment to universal legal capacity and the abolition of coercive interventions risks outpacing existing social infrastructures and may even weaken substantive rights in high-risk situations. Second, cross-agency coordination confronts poorly coordinated departments and unclear accountability. Third, monitoring and data-driven oversight remains difficult to operationalize in many jurisdictions due to limited authority, resources, and data capacity. In response, this article advances three corresponding recommendations. WHO could strengthen the evidence base for rights-based mental health reforms by drawing more systematically on CRPD state reporting, committee reviews, while facilitating cross-national research to support the effective implementation of the Guidance. Regarding the whole-sectoral coordination, a phased and procedurally oriented approach may offer a more realistic starting point. International assessment mechanisms could supplement domestic monitoring by providing comparative legal assessments, supported by structured expert training and the inclusion of people with lived experience. While these challenges temper expectations of immediate transformation, the 2023 Guidance nonetheless makes a significant contribution by reorienting mental health law toward rights–based approaches and providing a durable normative foundation for future reform.

## 1 Introduction

One of the dominant concerns of mental health laws globally is how to strike an acceptable balance between therapeutic care, individual liberty, and possibly public safety in some jurisdictions. Interventions such as involuntary hospitalization, compulsory treatment, seclusion, and restraint have long been treated as unavoidable features of legal regulation. This logic has shaped the basic architecture of mental health legislation in many jurisdictions since the late twentieth century and has informed international policy guidance for decades (1, 2). Over the past twenty years, however, developments in international human rights law have placed this framework under sustained pressure. Most notably, the adoption and implementation of the United Nations Convention on the Rights of Persons with Disabilities (CRPD) (3) has unsettled foundational assumptions that once structured mental health law. The CRPD rejects disability-based restrictions on rights as a matter of principle (4), which calls into question the exceptionalism that has historically underwritten mental health legislation. In this trend, mental health is no longer approached solely as a medical or welfare concern, but increasingly appears as a site of rights, citizenship, and social justice, prompting renewed scrutiny of the legitimacy, purposes, and limits of legal intervention in this domain. Situated at the intersection of psychiatric knowledge, international human rights norms, and domestic legal reform, the mental health legislative guidance issued by the World Health Organization (WHO) articulates standards that reflect evolving professional and ethical understandings while simultaneously shaping how mental health law is imagined and reformed across national contexts. Therefore, the changes in the WHO guidance document offer a particularly revealing lens through which broader shifts in the normative foundations of mental health law may be traced.

Existing scholarship has engaged extensively with the implications of the CRPD for mental health law, much of which concentrates on discrete institutional controversies in different specific countries, including the legality of involuntary hospitalization, the assessment of legal capacity, or the feasibility of supported decision making (5–9, 33, 34). Very limited attention has been paid to whether international mental health legislative guidance has undergone a fundamental normative reorientation, or whether the direction and scope of the changes raise concerns about radicalism or bias (10). Longitudinal analysis remains limited with respect to how authoritative policy documents articulate changing legislative philosophies, value commitments, and governance logics over time. The existing literature does not directly call for a longitudinal comparative analysis of WHO’s mental health legislative guidance. Nonetheless, the need for such analysis is implicitly suggested by the central role that both WHO legislative frameworks and the CRPD now play as normative benchmarks in evaluating national mental health laws and policies (26–29). Yet, whether those benchmarks have undergone fundamental reorientation remains unexamined. Some earlier scholarship compared the CRPD with the 2006 WHO Resource Book on Mental Health, Human Rights and Legislation and identified important normative tensions and shifts (1). However, those analyses were conducted prior to the publication of the WHO’s latest *Guidance and Practice* issued in 2023. As a result, there has been little systematic examination of whether WHO legislative guidance has undergone a fundamental normative reorientation in the post-CRPD era.

This article seeks to address that gap through a comparative analysis of two significant WHO guidance documents: the *WHO Resource Book on Mental Health, Human Rights and Legislation* issued in 2006 (11) (hereafter “2006 Book”), and *Mental Health, Human Rights and Legislation: Guidance and Practice* recently issued in 2023 (12) (hereafter “2023 Guidance”). By examining their respective approaches to involuntary hospitalization, legal capacity and decision making, service organization, participatory governance, and accountability mechanisms, the article traces how the normative grammar of international mental health law has evolved. The 2006 Book remained committed to a legislative philosophy that endorsed stand-alone mental health statutes and treated coercion as inevitable, seeking to discipline and justify it through procedural safeguards rather than questioning its legitimacy. This approach was coupled with a balancing conception of rights that placed individual interests alongside public safety and accepted restrictions on liberty and legal capacity under specified conditions, such as a diagnostic threshold, dangerousness to self or other, and necessity of treatment. The 2023 Guidance, published seventeen years after the 2006 Book, responds to more than a decade of normative and institutional developments following the entry into force of the CRPD in 2008 (3). The CRPD fundamentally recasts disability as a matter of human rights and equality rather than impairment or risk by breaking the long-standing assumption that coercion could be rendered legitimate through procedural regulation. This shift generated sustained pressure on international organizations, including the WHO, to reassess guidance that continued to accommodate coercive mental health regimes. Despite its non-binding nature, the 2023 Guidance marks a substantial advance from the 2006 Book from a biomedical model toward a more advanced, flexible human rights model required by CRPD (2). In the 2023 Guidance, Chapter 1 sets out the core legal principles and legislative ethos underpinning this shift, reframing mental health legislation through a person-centered, rights-based approach that foregrounds equality, autonomy, and community inclusion. Chapter 2 articulates specific legal and institutional components of rights-based mental health systems, including legal capacity, informed consent, community-based services, and participation and accountability. Chapter 3 addresses the implementation of rights-based reform by outlining participatory, evidence-informed legislative processes and institutional arrangements aimed at ensuring feasibility, sustainability, and ongoing evaluation. Overall, the Guidance aims to reorient the core values of mental health and actively encourages the WHO’s member states to adopt its guiding provisions for rights-based legislative reform.

The central question explored in this article is not only how international guidance has been updated or refined, but also how it reflects a deeper paradigmatic transformation. The article demonstrates that WHO’s legislative guidance on mental health has moved beyond a framework that justified coercive intervention through procedural safeguards. It advances a human right-centered approach that rejects coercion as a governing principle and reconstructs legal capacity and service delivery around support instead of substitution. Through systematic comparison, the article shows how the CRPD has reshaped the justificatory foundations of mental health law. The contribution of this study lies in its structural perspective. Rather than isolating individual doctrines or practices, it clarifies the logic of transition from regulating coercion to rejecting it as a normative anchor and offers an analytical framework for understanding how human rights-based paradigms are transforming legislative design and service systems across diverse legal contexts. The article proceeds as follows. The first section compares the legislative philosophies and value commitments reflected in the 2006 and 2023 documents. The second section analyzes the challenges faced by the domestic implementation of WHO 2023 Guidance in general, including goal setting, cross-departmental cooperation, and supervision mechanisms, and proposes corresponding response suggestions. The final discussion section synthesizes the findings and reflects on the implications of the 2023 Guidance for the future trajectory of mental health law reform.

## 2 Method

This article adopts a qualitative text analysis of two WHO guidance documents on mental health legislation: the *Resource Book on Mental Health, Human Rights and Legislation* (2006) and *Mental Health, Human Rights and Legislation: Guidance and Practice* (2023). These documents were selected as the only two comprehensive guidance frameworks issued by the WHO for the purpose of assisting mental health law making and reform at domestic level. Their seventeen-year interval spans the entry into force of the CRPD, providing a meaningful temporal window through which to examine normative change. Text analysis allows us to clearly and directly capture the development and changes in the WHO legislative framework. The analysis was conducted through systematic close reading of both documents in full. Attention was paid to identifying areas in which the 2023 Guidance develops, reframes, or reinterprets positions articulated in the 2006 Resource Book. Five thematic dimensions were then identified for focused comparison: involuntary admission; legal capacity and decision-making; seclusion and restraint; service user participation and oversight mechanisms; and community-based service models. These five dimensions were identified through preliminary full-text reading of both documents, capturing the clearest and most significant reorientation between WHO’s 2006 and 2023 legislative framework. They also correspond to the domains in which the shift toward a CRPD-aligned human rights framework is most visible and conceptually consequential. For each dimension, the normative positions, underlying justificatory logics, and institutional arrangements articulated in each document were examined and compared. The CRPD and its interpretive outputs, including relevant General Comments, served as the primary normative reference framework guiding the analysis. This study is limited to the textual and normative content of the two WHO documents. It does not assess domestic legislative outcomes or implementation experiences across individual jurisdictions.

## 3 The evolution of WHO guidelines: from 2006 to 2023

This section traces the evolution from the WHO’s 2006 Book to the 2023 Guidance, highlighting a fundamental reorientation in how mental health law is conceptualized, justified, and operationalized. The 2006 Book reflects an approach that sought to reconcile coercive psychiatric practices with human rights primarily through legalization, procedural safeguards, and professional regulation. By contrast, the 2023 Guidance marks a decisive shift toward a rights-based paradigm grounded in the CRPD. Regarding involuntary admission, legal capacity and decision-making, seclusion and restraint, participation and accountability, and community-based service, etc., the 2023 Guidance moves away from conditional acceptance and procedural containment of coercion, toward its principled rejection and replacement with support-based, person-centered alternatives. **Table 1** presents this shift in a clearer and more concise way. This transition reflects not merely incremental policy refinement but a deeper transformation in the understanding of autonomy, equality, and state responsibility, shifting from managing risk and regulating deviation to enabling legal agency, social inclusion, and meaningful participation for persons with psychosocial disabilities, a term increasingly used following the adoption of the CRPD. In this sense, the 2023 Guidance repositions mental health reform as a project of redistributing power, reconfiguring institutions, and re-embedding mental health governance within the broader human rights architecture, representing structural progress from the 2006 framework.

**Table 1:** Summary of comparison between 2006 Book and 2023 Guidance.

Thematic dimension	2006 Book	2023 Guidance
Involuntary Admission	Exceptional but legitimate regulatory tool. Triple threshold: mental disorder + dangerousness + therapeutic purpose. Procedural safeguards as source of legitimacy.	Call for the elimination of coercion. Diagnosis cannot justify detention. Procedural refinement cannot legitimate discriminatory coercion. Community services and crisis support as alternatives.
Legal Capacity and Decision-Making	Distinction between juridical “capacity” and functional “competence”. Substitute decision-making permissible upon capacity assessment. Scope confined by procedural safeguards, not questioned in principle.	Presumption of universal legal capacity. Replacement of substitute decision-making. Supported decision-making based on individual’s “will and preferences”. Advance directives elevated as extensions of legal capacity.
Seclusion and Restraint	Conditionally permitted as “last resort” in emergencies. Justified by urgency and necessity. Oversight limited to internal institutional review.	Call for prohibition of seclusion and restraint. Redefined as potential human rights violations. De-escalation and crisis support as alternatives. Independent monitoring and remedy mechanisms.
Service User Participation and Oversight	Participation acknowledged as supplementary, not rights-based. Professional-led, administratively centralized governance. Oversight narrowly focused on legality of involuntary measures.	“Nothing about us without us”. Full and effective involvement in policy, service design, and monitoring. Lived experience as useful resource. Participation institutionalized and made accountable.
Community-Based Service Models	Community services desirable but subordinate to psychiatric hospitals. Medically oriented: mental distress as health condition requiring professional intervention.	Deinstitutionalization as center of reform. Person-centered, rights-based community services. Peer-led services and non-coercive crisis support. Recovery goal: self-defined meaning, not cure.

### 3.1 Progressive evolution in involuntary admission

In the 2006 Book, involuntary admission was not presented as a desirable form of mental health intervention, yet it was accepted as a practice that could not be entirely avoided in certain circumstances. The 2006 Book set out in detail for its application conditions, substantive thresholds and procedural requirements (pp. 46-52), the underlying rationale of which is to constrain the scope and intensity of state interference with personal liberty through legalization and proceduralizing. At the application level, the 2006 framework treated involuntary admission as an exceptional but legitimate regulatory tool within mental health systems, premised on the assumption that coercion, while regrettable, could be rendered compatible with human rights through careful legal design. At the level of substantive standards, the 2006 framework held that involuntary admission could be justified only where “mental disorder”, “serious likelihood of immediate or imminent danger and/or ‘need for treatment’”, and “a therapeutic purpose” need was simultaneously present (pp. 49-50).

On the procedural level, it emphasized independent review and appeal mechanisms, explicit time limits, and regular, time-bound reassessment, aiming to prevent abuse of coercive powers and to mitigate long-standing problems of medical dominance and arbitrary detention in the mental health field (pp. 50-52). As the CRPD acquired a central position within the international human rights regime, the limits of this strategy of legitimizing coercion through procedural safeguards became increasingly visible. Article 14 of the CRPD makes a further step to emphasize that the existence of a disability can never justify deprivation of liberty, a position that is further reinforced by the Committee’s interpretation of legal capacity under Article 12 (13), and Article 25 also affirms that medical interventions must be grounded in free and informed consent, which fundamentally undermine the normative foundation of the 2006 framework that sought to justify involuntary hospitalization by combining mental disorder diagnoses, assessments of dangerousness, and procedural protections.

Building on these developments brought by the CRPD, the 2023 Guidance adopts “zero coercion policy” to involuntary hospitalization, which explicitly states that such practices raise serious human rights concerns and may lead to severe forms of abuse (pp. 66-67). In the dimension of substantive standards, the 2023 Guidance further develops and revises the earlier approach by clearly asserting that a diagnosis of mental disorder should never constitute a legal basis for hospitalization or detention, and “all mental health interventions and services shall be provided on the basis of persons’ free and informed consent” (p. 68). This position directly reflects Article 14 of the CRPD that rejects disability as a justification for deprivation of liberty. The 2023 Guidance further refuses to treat “dangerousness” as a trigger for coercive intervention. Instead, it emphasizes de-escalation strategies and conflict-resolution mechanisms aimed at reducing reliance on law-enforcement responses and other coercive measures. This shift resonates with the CRPD’s critique of preventive detention and risk-based confinement, which has emphasized that predictions of future harm cannot override the principles of legal capacity, equal recognition before the law, and non-discrimination.

A corresponding transformation can also be observed in the approach to procedural design. As noted above, the 2006 Book placed significant weight on procedural safeguards as a means of limiting coercion, assuming that procedural justice could supply a degree of legitimacy to involuntary hospitalization. By contrast, the 2023 Guidance makes explicit that procedural refinement alone cannot legitimize coercive measures that are discriminatory in substance. It rather calls for community-based services, advance directives, supported decision-making, peer support, and appropriate crisis support as alternatives to involuntary intervention (p. 67), as a response to Article 12 of the CRPD. Even in emergency situations, the 2023 Guidance still insists that priority should be given to voluntary and supportive responses, including crisis support services, instead of resorting to coercive measures (p. 67).

### 3.2 The transformation of legal capacity and decision-making authority

The 2006 Book drew a conceptual distinction between “capacity” and “competence”. “Capacity” was treated as a juridical concept referring to a person’s recognition before the law and their legal and social standing, whereas “competence” was framed as a health-related concept concerning an individual’s functional abilities and cognitive functioning (pp. 39-40) which usually involves understanding, retaining, and weighting relevant information. The 2006 Book acknowledges that psychosocial or intellectual impairments might, in certain contexts, affect an individual’s ability to make decisions or to participate effectively in decision-making processes. It therefore accepted the permissibility of substitute decision-making when a person was assessed as lacking capacity or as having severely impaired decision-making abilities. Although the 2006 Book sought to confine the scope of substitute decision-making through stringent assessment criteria, procedural safeguards, and periodic review, its underlying position remained that legal capacity could, in practice, be restricted or transferred on the basis of mental capacity evaluations (p. 40).

The 2023 WHO Guidance marks a fundamental shift from such understanding of restricted legal capacity toward a presumption of universal legal capacity. This position shift reflects a deeper and more comprehensive understanding of legal capacity under Article 12 of the CRPD, which affirms the universality and relevance of legal capacity across all areas of life, including mental health-related decision-making. It affirms that all people, in all circumstances, are entitled to “be recognized as a person before the law and as having legal capacity on an equal basis with others; and (b) be provided access to the freely chosen support they may require to exercise their legal capacity” (p. 47). Decision-making difficulties thus should be addressed through the provision of appropriate institutional and social support, rather than through the removal or transfer of decision-making rights (p. 46). More specifically, the 2023 Guidance advances the model of supported decision-making, under which decision-making authority remains with the individual, while the role of external actors is limited to “assistance”. Decisions are to be guided by the person’s own “will and preferences”, prioritizing their expressed choices rather than third parties’ assessments of the individual’s “best interests” (p. 53). The latter involves consideration of a person’s welfare with all relevant contextual factors but is usually determined in a paternalistic fashion by someone other than the person concerned, i.e., a family member or medical professional.

In the treatment of advance decision-making tools, the 2006 Resource Book only made several references to it, without offering a systematic account of their legal status, conditions of application, institutional function, etc. The 2023 Guidance significantly elevates the role of advance planning and advance directives as core components of supported decision-making and regards them as extensions of disabled individuals’ legal capacities across time. It means that, through advance planning, individuals are able, when they have full expressive capacity, to set out clear preferences for future decision-making contexts, preferences that should then receive priority in subsequent supported decision-making processes (p. 62). This approach further consolidates the centrality of individual will and preferences throughout the mental health decision-making regime.

### 3.3 Shifting attitudes toward seclusion and restraint

In the 2006 Book, seclusion and restraint remained conditionally acceptable. It stated that, as a general principle, restrictive measures such as seclusion and restraint should not be applied, yet it allowed their use in narrowly defined emergency situations under professional supervision where they were considered “the only means available” of preventing immediate and serious harm to the person concerned or to others (p. 65). This regulatory logic, centered on urgency and necessity, nonetheless assumed that seclusion and restraint possessed a degree of institutional legitimacy within mental health practice. The 2023 Guidance made substantial progress by calling for the comprehensive prohibition of all forms of seclusion and restraint, including physical isolation, mechanical restraint, chemical restraint, and any practice that restricts freedom of movement (pp. 72-73). This stance aligns with Article 14 of the CRPD, which prohibits deprivation of liberty based on disability, and Article 15, which guarantees freedom from torture and from cruel, inhuman, or degrading treatment. This means that the 2023 Guidance has redefined the “medicalized” coercive measures as potential human rights violations, which has been well acknowledged in both high-income and low- and middle-income countries (30) and attracting growing studies in developing policies and best practice to minimize coercion in mental health settings (31).

Regarding the alternatives to coercive measures, the 2006 Book focused on how to restrict the conditions and procedures for the use of isolation and restraint rather than finding milder alternatives (pp. 72-73). While the 2023 Guidance, as a response to Article 19 of the CRPD on independent living and community inclusion, highly emphasized the alternative measures such as de-escalation strategies, crisis support services, comfort rooms, and calming environments, aiming to fundamentally decrease the reliance on seclusion and restraint (p. 72). The focus shift from risk containment (2006 Book) to the provision of support (2023 Guidance) embodies the CRPD’s broader movement from “control-oriented care” toward “support-based services”, in which coercive measures are no longer treated as necessary tools of crisis management.

Changes in oversight and accountability further reinforce this transformation. The 2006 Book relied primarily on case records and internal institutional reviews. It only required detailed documentation of each instance of seclusion or restraint and subjected such use to procedural review by designated bodies, with the aim of ensuring compliance with the principles of “last resort” and “minimum necessity” (p. 64). While the oversight duties under this model remained only within mental health institutions. The 2023 Guidance, by contrast, substantially strengthens the institutional architecture of supervision and accountability. It calls for systematic recording and reporting of all incidents of seclusion and restraint, their inclusion within independent monitoring mechanisms, and, where appropriate, investigation of specific incidents and responsible actors, alongside the establishment of avenues for remedy and compensation (pp. 101-102). The 2023 Guidance further requires relevant legislation to ensure the institutional, financial, and political independence of these monitoring bodies. This framework responds to Article 33 of the CRPD, which mandates the establishment of independent monitoring mechanisms, and elevates seclusion and restraint from matters of professional discretion to issues of broader public accountability as human rights concerns.

### 3.4 Evolving service users participation and oversight mechanisms

In the 2006 Book, service users’ participation mechanisms were embedded in a professional-led and administratively centralized model of governance. Service users’ involvement was acknowledged only in a limited and largely procedural manner as a supplementary element rather than as an entitlement grounded in rights. Oversight mechanisms were also narrow in scope, focusing primarily on the legality of involuntary measures and seeking to prevent only the most extreme or manifest procedural abuses. The 2006 framework was basically concerned with ensuring that existing mental health interventions operated in a legally defensible manner, rather than reconfiguring power relations in a more humane and egalitarian direction.

The 2023 Guidance moves decisively beyond the 2006 framework by incorporating participation, empowerment, and accountability as integral components of mental health governance, in line with Articles 4(3) of the CRPD, which require the active involvement of people with disabilities and their representative organizations in public decision-making and oversight processes. People with psychosocial disabilities are no longer positioned merely as recipients of policy effects but are recognized as rights-bearing subjects entitled to participate in public decision-making processes that directly affect their bodies, lives, and liberty. Through the consistent emphasis of the principle “nothing about us without us,” the 2023 Guidance gives concrete expression to Articles 4 (3) and 29 of the CRPD on the participation of persons with disabilities in public affairs. In contrast to the near absence of people with lived experience in the 2006 framework, the 2023 Guidance explicitly requires their participation in policy formulation, service design, and monitoring and evaluation processes. “Persons with lived experience” refers to individuals who have direct, personal experience of mental health conditions and the associated social, legal, or service systems, rather than solely professional or observational knowledge. The 2023 Guidance requires legal and regulatory frameworks to ensure the “full and effective involvement” of people with lived experience and their representative organizations in “public decisions concerning issues such as mental health provision, legislation, policies, strategies and action plans” (p. 97). Lived experience constitutes a form of knowledge that can complement professional expertise and, once incorporated into professional frameworks, has the potential to challenge the paternalistic assumptions embedded within them (32). Participation is further institutionalized and made accountable through procedural design, including institutionalized consultation processes, requirements of representativeness, and safeguards to ensure meaningful engagement.

### 3.5 Transformation of community-based service models

In the 2006 Book, community-based mental health services were presented as a desirable policy direction, yet they were still positioned as subordinate to an institutional system centered on psychiatric hospitals. The role of community institutions was only confined to reducing costs, improving accessibility, avoiding unnecessary hospitalization, etc. Its underlying legislative logic remained grounded in a medically oriented understanding of mental distress, treating it as a “health condition” to be addressed by professional medical intervention (p. 45-46). By contrast, the 2023 Guidance seeks to break with this medically dominated model of rehabilitation and to reorient mental health services toward “‘the whole person’, not their mental health diagnosis” (p. 90). It advances deinstitutionalization as the center of mental health reform and emphasizes that “person-centered and rights-based community” mental health and support services should be delivered primarily and preferentially within community settings. In response to Article 19 of CRPD, i.e., the right to live independently and be included in the community, it outlines concrete implementation approaches including legal reform, the development of community-based alternatives, the reallocation of resources, and transitional safeguards for the rights of individuals currently residing in institutional settings. Drawing on the UN CRPD Committee’s *Guidelines on Deinstitutionalization*, deinstitutionalization, understood to encompass the provision of sufficient and high-quality community-based services, is framed not merely as a policy preference, but as a necessary condition for realizing equal citizenship and preventing the structural exclusion produced by long-term institutional residence (39).

In terms of service design, the 2023 Guidance emphasizes rehabilitation should be oriented toward autonomy, participation, and social inclusion rather than treatment or correction alone. It therefore requires the development of “peer-led” and “peer-run” services, non-coercive crisis support, community-based living arrangements, and integrated support delivered by multidisciplinary teams. The introduction of peer workers with lived experience is also encouraged to decenter diagnostic authority and reduce the dominance of clinical control within service provision. The final recovery goal of the 2023 Guidance is not “curing” or making people “healthy” or “normal again”; rather on “supporting people to identify what recovery means to them” (p. 90). In this way, people with mental disabilities are supported in gaining or regaining control of their identity and life, have hope for the future, and live a life that has meaning for them.

## 4 Challenges and actionable recommendations

### 4.1 Implementation challenges

The 2023 Guidance adopts a more ambitious orientation with a significantly higher compliance threshold for mental health legislation. Its core aspiration is to move mental health reform systematically beyond a biomedically centered model toward a rights-based framework. Some of the reforms it advances, including deinstitutionalization, comprehensive cross-sectoral coordination, oversight and accountability mechanisms, and the redistribution of financial resources, etc., might place demands that are difficult to be accommodated within current situations. The prevailing administrative arrangements, accountability structures, and risk-governance tools remain deeply embedded in a governance logic organized around departmental boundaries, professional control, and the maintenance of public order. Existing research has shown that even in states that have ratified the CRPD for many years, domestic legal frameworks and practices continue to exhibit highly uneven levels of compliance with the CRPD’s core obligations, revealing persistent structural gaps (2, 14, 36–38). Although the 2023 Guidance is clearly shaped by the normative commitments of the CRPD, it is foreseeable that the barriers it encounters are no less significant than those faced by the CRPD itself. The following section examines three key challenges that arise from this implementation context.

One of the most fundamental challenges in the domestic implementation of the 2023 Guidance lies in the tension between progressive human rights norms and current mental health governance structures. Its provisions on legal capacity and personal liberty clearly build on the normative commitments of the CRPD and seek to advance them further. However, skepticism has already emerged around the legal capacity framework articulated in Article 12 of the CRPD and its General Comment No. 1 (13). Critics have argued that the CRPD Committee’s interpretation may generate unintended consequences, including potential infringements on the right to the highest attainable standard of health, access to justice, the right to liberty, and even the right to life (15, 16). There may well be the case for the 2023 Guide. For example, the 2023 Guidance advocates replacing capacity-based substitute decision-making regimes with a presumption of universal legal capacity supported through supported decision-making arrangements. It also explicitly calls for the gradual elimination of involuntary hospitalization and treatment based on mental disability. However, this approach may, if implemented in the absence of corresponding social supports and alternative governance mechanisms, undermine the substantive protection of persons with psychosocial disabilities (15). On the one hand, it may leave mental health systems unable to respond effectively to acute mental health crises or situations involving a high risk of serious self-harm or harm to others (17). The withdrawal of timely and necessary intervention may in practice weaken individuals’ enjoyment of the highest attainable standard of health. On the other hand, when medical systems are prohibited from intervening while risks remain unresolved, states often shift toward reliance on criminal law, policing, or ordinary detention measures. As a result, deprivations of liberty that might otherwise have been constrained through medical oversight and procedural safeguards are transformed into more punitive and de-medicalized forms of control, to the detriment of both personal liberty and access to judicial remedies. In practice, involuntary hospitalization, compulsory treatment, and guardianship regimes have long functioned not only as part of mental health law in many jurisdictions, but as central institutional tools for risk management and the maintenance of public order (18). As several critics have observed, the insistence on the absolute recognition of legal capacity may, in certain contexts, generate outcomes that run counter to its original objectives (15).

The Challenges of Cross-Sectoral Governance. The 2023 Guidance emphasizes that mental health reform is not an “internal matter” of the health sector, but a “cross-cutting human rights issue that requires whole-of-government approach” (p. 106) including housing, social security, employment, education, justice, and anti-discrimination etc. However, such cross-sectoral coordination has proven far from straightforward. Public governance continues to operate through sectoral specialization and vertical accountability structures. Legal mandates, budgetary arrangements, performance indicators, and responsibility allocations remain fragmented across departments, with few stable horizontal coordination mechanisms in place (14). These coordination difficulties are particularly acute in the mental health field. In the absence of clearly articulated interdepartmental obligations and integrated responsibility frameworks, mental health reforms in practice are often forced to fall back on the health system as a residual provider, requiring it to assume social support functions that lie well beyond the proper scope of medical care (19). Prior experiences with the domestic implementation of the CRPD demonstrated that insufficient cross-sectoral coordination is not merely a matter of isolated policy failure, but a structural constraint (33, 36). As reports by the Office of the United Nations High Commissioner for Human Rights (OHCHR) have noted, rights would only remain in symbolic affirmation if the operational logics of housing, social welfare, and employment systems are not adjusted in parallel (20). Hence, the multi-sectoral responsibility structure articulated by the 2023 Guidance poses a substantive challenge to existing modes of state governance. In the absence of institutionalized coordination mechanisms, unified accountability frameworks, and tools for resource integration, its reform demands are highly susceptible to implementation gaps characterized by dispersed responsibilities without effective ownership. The result is a recurring structural disjunction in which legislative ambitions are normatively progressive, yet their practical effects remain limited.

Limited Enforceability of Accountability Mechanisms. The 2023 Guidance explicitly identifies monitoring, data collection, and independent oversight mechanisms as core components that should be embedded in mental health legislation. It emphasizes the independent monitoring bodies should align their functions with the domestic oversight frameworks, as required by Article 33 of the CRPD. However, comparative studies indicate that many states have either not established such bodies, or have done so only in a formalistic manner, without providing them with adequate statutory powers, financial resources, or investigative capacity (21). Oversight bodies also face significant barriers to accessing closed or highly securitized service settings, which severely constrain their ability to conduct sustained, on-site, and effective monitoring of coercive measures, seclusion practices, and involuntary interventions (22). As a result, the model of “external and independent oversight” envisioned by the Guidance is easily reduced in practice to a largely “rubber stamp” (22). The 2023 Guidance further emphasizes the establishment of indicators and data collection mechanisms relating to service utilization, system performance, and reform outcomes, treating information accessibility and transparency as preconditions for meaningful oversight and accountability. At the level of domestic implementation, however, data collection itself frequently encounters multiple technical and institutional obstacles. These include the absence of standardized indicators, unresolved tensions between data collection and privacy or data protection regimes, and a lack of interoperability between health systems and social service systems (21). Such gaps in data capacity make it difficult to conduct sustained, comparable, and accountable monitoring. The Guidance’s institutional vision of data-driven accountability mechanisms still face considerable barriers.

### 4.2 Policy recommendations

Considering the three institutional obstacles identified above, this article attempts to give a set of corresponding recommendations.

#### 4.2.1 Recommendation 1: strengthen the evidence base for rights-based reform

Given the gap between the WHO’s ambitious provisions on legal capacity and personal liberty and existing domestic realities, the WHO’s role should extend beyond articulating reform ideals. It should also foster systematic evidence based on how rights-based mental health reforms operate in practice. Since the entry into force of the CRPD, a large body of state reports, committee reviews, and independent shadow reports have documented progress in rights implementation, as well as institutional arrangements, obstacles, and policy outcomes. Drawing more fully on the practical experiences revealed in CRPD state reporting could make normative guidance more attentive to feasibility, sequencing, and institutional capacity. Beyond the data generated through CRPD review mechanisms, the WHO might further be encouraged to develop a more robust evidence base on the practical consequences of implementing these reforms, alongside the advancement of normative reform ideals. One possible approach is for the WHO to coordinate cross-national comparative research examining the effects inspired by the 2023 Guidance after a period of implementation, with particular attention to outcomes in rights protection, crisis response capacity, service accessibility, and patterns of risk redistribution across different governance contexts. In terms of evidentiary standards, these studies should also explicitly recognize lived experience as an indispensable source of knowledge, incorporating the experiential accounts of people with psychosocial disabilities and their support networks alongside institutional outcomes. Only by integrating experiential evidence with empirical data can it be assessed, in a context-sensitive manner, whether reforms that appear normatively progressive have in fact improved the realization of rights across diverse institutional settings.

#### 4.2.2 Recommendation 2: adopt a phased approach to cross-sectoral coordination

Although the 2023 Guidance promotes a “whole-of-government” reform, it seems to overestimate states’ short-term capacity for deep institutional coordination and structural adjustment. A phased, low-threshold cooperative framework may offer a more realistic starting point for reform. It does not need to extend across all relevant policy domains. Instead, priority may be given to a limited set of institutional interfaces that are decisive for the outcomes of mental health reform, for example, housing, social welfare provision, and employment (40, 41). Similarly, regarding the abolition of coercive measures, a more feasible reform pathway may lie not in immediate and comprehensive abolition, but in aligning legal reform with a gradual reconfiguration of resource allocation and service structures. This may include placing constraints on new investments in large-scale, segregated institutions, while progressively redirecting resources previously devoted to coercive practices and institutional expansion toward supported decision-making frameworks, peer support networks, community-based crisis response, and integrated social support services. Another way to address the challenges of cross-sectoral coordination is to begin with procedural forms of collaboration, including consultation, referral, joint assessment, and information sharing, etc. These procedural designs could gradually weaken institutional silos without immediately altering core departmental functions, thereby creating space for incremental learning and future integration of responsibilities. Such a phased, procedural approach is more likely to realize the ambitions of the Guidance within existing governance structures over time.

#### 4.2.3 Recommendation 3: supplement domestic monitoring with international assessment mechanisms

To address the challenges of independent domestic monitoring, a more institutionally realistic approach is to first introduce external and comparative assessment mechanisms at the global level. WHO could commission independent experts to use the 2023 Guidance’s checklist to systematically assess national mental health legislation and make the results publicly available. This kind of comparative legal evaluation would not replace domestic oversight mechanisms but could serve to increase transparency and create reputational incentives for reform—a form of “soft accountability” that has been used in other international legal and human rights contexts (23, 35). Within this international monitoring architecture, people with lived experience should also be accorded formal institutional roles to supply experts with empirical feedback. To further ensure that such assessments are robust and context sensitive, WHO could refer to its 2006 Book which outlined the training for mental health professionals and related stakeholders. Specifically, WHO could support the training of cohorts of independent legal and policy experts — including those from national human rights institutions, civil society, and relevant professional communities — on rights-based approaches to mental health law. The WHO’s *QualityRights Initiative* has also produced a wide range of training and advocacy resources, which could be more fully utilized by states (24). Once trained, these experts would be well placed to undertake independent assessments of national legislation in accordance with the 2023 Guidance’s standards. For example, China’s former Ministry of Health commissioned WHO experts and consultants to provide training on mental health legislation (25). By linking international legal assessment with a structured expert training pipeline, this approach not only enhances the quality and legitimacy of comparative evaluations but also strengthens the capacity of domestic actors to engage with rights-based legal reform processes.

## 5 Discussion

This article examined nearly two decades of evolution in the WHO’s mental health legislative guidance from 2006 to 2023, situates these changes within broader international human rights discourse and psychiatric governance. While existing comparative and international scholarship on mental health law has largely centered on the CRPD and its domestic implementation, less attention has been paid to the WHO’s guiding position within mental health law. This article shows that WHO’s guiding clauses, though not legally binding, function not merely as a set of technical recommendations, but as an evolving framework through which legal and professional principles are continuously negotiated.

The comparison shows that WHO’s 2023 Guidance represents a substantial advance from its 2006 framework. In core principles, the WHO has moved from a biomedical orientation toward an explicitly rights-based model, from reliance on specialized mental health legislation toward integration within general legal frameworks, and from balancing protection and control toward comprehensive rights protection. These shifts are accompanied by fundamental changes in legal architecture. The 2023 Guidance promotes deinstitutionalization and community-based services, rejects coercive interventions, supports decision-making grounded in universal legal capacity, etc. Implementation strategies have likewise evolved. It increasingly emphasizes participation, the leadership of persons with lived experience, and cultural transformation within mental health systems. These developments reflect the significant influence of the CRPD, especially the human rights model of mental health law. This transformation represents a deeper shift in values and problem framings, reflecting an expanded understanding of mental health as a rights-based issue.

The normative trajectory documented in this article is not merely theoretical. Emerging evidence from both low- and middle-income countries and high-income countries suggests that the paradigm shift reflected in WHO’s guidance has begun to find expression in domestic legislative processes, though the pace and depth of adaptation vary considerably across jurisdictions. In the African context, research has shown that states including Ghana, Nigeria, Kenya, Egypt, and Cabo Verde have enacted or revised mental health legislation in ways that increasingly engage with human rights standards drawn from the CRPD and international guidance frameworks (27, 28). For example, Ghana’s Mental Health Act 2012 has been recognized as a progressive legislative instrument within the African region, incorporating provisions on voluntary treatment, independent oversight, and service user rights that align with the normative direction charted by WHO guidance. In Asia, China’s Mental Health Law 2012 and its subsequent implementation represents a partial but significant legislative shift: while retaining certain coercive mechanisms, its reform trajectory demonstrates incremental movement toward procedural safeguards and a reduction in restrictive measures—a pattern consistent with the transitional logic of the 2006 WHO Resource Book rather than the more transformative stance of the 2023 Guidance (7). India’s Mental Healthcare Act 2017 offers a more ambitious example, explicitly aligning its provisions with CRPD obligations and moving toward supported decision-making, informed consent requirements, and deinstitutionalization, which are broadly consistent with the rights-based orientation later articulated in the 2023 Guidance (24). In developed nations, ongoing legislative reviews in England, Wales, and Ireland have similarly grappled with the tension between existing coercive frameworks and the rights-based standards advanced by CRPD and WHO guidance, reflecting the contested institutional dynamics this article describes (1). Taken together, these country-level developments illustrate that the normative shift documented in this study is not merely a textual phenomenon. They also confirm that the implementation gap this article identifies between the progressive ambitions of WHO’s 2023 Guidance and the structural realities of governance is empirically observable across both LMICs and developed legal systems. A systematic comparative analysis of how specific jurisdictions have adapted their legislation in response to evolving WHO and CRPD standards remains an important avenue for future research.

Rights-based standards, however progressive, risk remaining symbolic if they are not matched by effective implementation strategies and feedback mechanisms. The central question, therefore, is not simply whether states adopt the language of the 2023 Guidance. It is whether the Guidance possesses sufficient operational capacity to shape practice. The capacity of the 2023 Guidance to catalyze substantial legal changes faces several challenges. First, some of its ambitious proposals sit uneasily with existing mental health governance structures. Its approach to legal capacity and the rejection of coercive measures often exceeds the short-term capacity of states. Second, while the Guidance emphasizes whole-of-government responsibility, many governance systems remain organized around sectoral mandates and vertical accountability. As a result, sustained cross-sectoral coordination is difficult to achieve in practice. Third, although independent oversight and data collection are identified as central to accountability, domestic monitoring bodies are often under-resourced. Many are formally established but substantively weak. In response to these challenges, this article advances a set of process-oriented and institutionally realistic pathways. WHO could play a stronger role in generating systematic evidence on how mental health reforms operate in practice, including through cross-national comparative research. Rather than pursuing comprehensive integration from the outset, cross-sectoral governance may be more effectively advanced through phased, low-threshold coordination at key institutional interfaces, such as housing, social welfare, and crisis response. Such coordination can be achieved primarily through procedural mechanisms. To address monitoring and accountability gaps, domestic oversight may be complemented by international assessment mechanisms coordinated by the WHO. In sum, despite its limitations and non-binding nature, the Guidance has the potential to influence current ways of thinking about mental health governance and to promote the gradual development of a more flexible, person-centered model.
